# Transcranial Magnetic Stimulation in Smoking Cessation: A Narrative Review of Neurobiological Mechanisms from Craving Modulation to Neural Circuit Restoration

**DOI:** 10.3390/brainsci16040392

**Published:** 2026-04-02

**Authors:** Dan-Alexandru Constantin, Denisa Bianca Cristina, Florin Gabriel Leașu, Andrada-Georgiana Nacu, Liliana Marcela Rogozea

**Affiliations:** Department of Fundamental, Prophylactic and Clinical Sciences, Faculty of Medicine, Transilvania University of Brasov, 500019 Brasov, Romania; englober@icloud.com (D.-A.C.); breniucbianca@yahoo.com (D.B.C.); florinleasu@unitbv.ro (F.G.L.); r_liliana@yahoo.com (L.M.R.)

**Keywords:** transcranial magnetic stimulation, repetitive transcranial magnetic stimulation, smoking cessation, nicotine dependence, craving, neural circuits, neuroplasticity, dorsolateral prefrontal cortex, neuromodulation, functional connectivity

## Abstract

**Highlights:**

**What are the main findings?**

• Randomized trials and meta-analyses suggest that active rTMS/deep TMS targeting prefrontal circuits (often DLPFC; deep coils including insula) reduces craving and cigarette consumption and may increase short-term abstinence versus sham.
• Neuroimaging findings link symptom improvement to changes in functional connectivity across reward, executive control, and salience networks, consistent with partial restoration of addiction-relevant circuit dynamics.

**What are the implications of the main findings?**

• Protocol features appear to matter for clinical impact: high-frequency stimulation (~10 Hz), adequate “dose” (≥20 sessions), and precise target localization are recurring elements in studies reporting stronger cessation-related outcomes.
• Future work should harmonize stimulation/outcome measures, include longer follow-up, and integrate moderators/biomarkers (e.g., baseline dependence, demographics, connectivity profiles) to personalize treatment and better separate true neuromodulation effects from placebo contributions.

**Abstract:**

**Background/Objectives:** Tobacco use is a leading cause of preventable death worldwide and is linked to major health and economic burden. Many smokers attempt to quit, yet long-term success rates with current medicines and counseling are still modest. Long-term nicotine exposure distorts brain systems involved in reward, craving, and self-control. These changes weaken inhibitory control and strengthen responses to smoking cues, which increases the risk of relapse. Transcranial magnetic stimulation (TMS) is a non-invasive technique that delivers magnetic pulses to specific cortical regions, most commonly the dorsolateral prefrontal cortex, to influence neural activity. This narrative review explored how transcranial magnetic stimulation may aid smoking cessation by acting on neural circuits linked to nicotine dependence. **Methods:** Five major databases were searched for studies published between 2015 and 2026. After removal of duplicates and screening, a total of 34 studies were included in this narrative synthesis. Randomized controlled trials, clinical studies, and neuroimaging investigations involving adults with nicotine dependence were included. A thematic narrative method was employed to synthesize findings due to the differences in study designs, protocols, and outcome measures. **Results:** TMS has been shown to attenuate cravings, decrease daily cigarette consumption, and decrease nicotine dependence in various studies. Several trials reported higher abstinence rates with active stimulation compared with sham treatment. Meta-analytic findings indicate stronger effects with 10 Hz stimulation and treatment courses of 20 sessions or more. Neuroimaging studies report changes in functional connectivity within reward, executive control, and salience networks, suggesting partial restoration of disrupted circuits. Treatment response varies according to age, educational level, baseline dependence, and stimulation parameters. **Conclusions:** These findings support transcranial magnetic stimulation as a promising brain-based approach for smoking cessation, while further well-designed trials with longer follow-up are still needed.

## 1. Introduction

Tobacco smoking is one of the major public health challenges worldwide. The World Health Organization estimates that there are more than 8 million deaths annually from tobacco use, including at least 1.2 million deaths from second-hand smoke exposure worldwide [1]. The economic burden of smoking is equally staggering, as healthcare expenses and productivity losses are estimated to be more than $1.4 trillion annually worldwide [2]. Despite decades of tobacco control efforts and education about the dangers of tobacco use and effective quitting strategies, there are still 1.3 billion tobacco smokers worldwide [3]. The current first-line pharmacotherapies used to aid in smoking cessation, which include nicotine replacement, varenicline, and bupropion, have been used together with either behavioral or cognitive-behavioral counseling [4,5]. However, the efficacy of these current pharmacotherapies is still relatively low, and this is reflected by the 20–35% long-term abstinence rates observed among those treated with the most effective pharmacotherapies after a year [6,7]. The rising rate of relapses among different groups and forms of treatments also highlights how strong nicotine addiction can be, emphasizing the need for treatments that target the neurobiological mechanisms sustaining tobacco dependence.

The limited success of current smoking cessation interventions can largely be attributed to the profound and constant neurobiological changes elicited by chronic nicotine exposure. Nicotine binds throughout the brain to nicotinic acetylcholine receptors and has especially potent effects on the mesolimbic dopamine pathway, activating the release of dopamine and other neurotransmitters that elicit the rewarding and reinforcing effects that maintain continued tobacco use [8]. Beyond these acute pharmacological effects, chronic nicotine exposure induces far-reaching neuroadaptive changes, including altered receptor expression, synaptic plasticity, and neuronal excitability [9]. Neuroimaging studies have reliably shown that nicotine-dependent individuals exhibit dysregulated activity and connectivity in several large-scale brain networks. The network formed by the ventral striatum, ventral tegmental area, and orbitofrontal cortex exhibits higher reactiveness when exposed to cues concerning smoking, as well as decreased reactiveness when presented with natural rewards [10,11]. Simultaneously, there is decreased reactiveness and connectivity of the executive control network, particularly in areas of the dorsolateral prefrontal cortex and dorsal anterior cingulate cortex, that negatively impacts cognitive control, decision-making, and inhibitory control [12,13]. The salience network, which has nodes in the anterior insula and in the dorsal anterior cingulate cortex, has abnormal interoceptive signal processing, increasing salience attribution to cues associated with drug use [14]. The distributed network alterations have provided a neurobiology that seeks to explain heightened cravings, diminished control mechanisms, and diminished ability to circumvent tobacco-related desires, thereby perpetuating a cycle of addiction.

Transcranial magnetic stimulation (TMS) has emerged as a promising non-invasive brain stimulation technique that could be of value in treating various neuropsychiatric conditions, including resistant forms of major depression, obsessive–compulsive disorder, and substance use disorders [15,16]. The basic principle is to make use of a coil that is placed on the scalp to transmit brief high-intensity magnetic fields. These fields induce electrical currents in the cortex that depolarize the membranes of the neurons [17]. When administered repetitively in structured protocols, TMS can produce lasting alterations in cortical excitability and plasticity that persist well beyond the stimulation session itself [18]. Generally, high-frequency rTMS increases cortical excitability and enhances synaptic efficacy due to processes mimicking long-term potentiation, whereas low-frequency stimulation suppresses cortical activity through processes more similar to long-term depression [19,20]. More recently developed protocols include theta burst stimulation, which delivers bursts of high-frequency stimulation at intervals corresponding to the brain’s theta rhythm, thus achieving robust plasticity induction in shorter treatment sessions [21]. Deep TMS uses special coil geometries, like the H-coil, to go deeper and stimulate larger volumes of the cortex, including deeper structures [22]. Thus, the treatment of addiction with TMS is based on the assumption that targeted neuromodulation of key nodes within addiction-relevant neural circuits can reinstate functional balance, enhance cognitive control, and reduce pathological craving responses.

The rationale for applying TMS to smoking cessation specifically derives from converging evidence that the prefrontal cortex, particularly the dorsolateral prefrontal cortex, is important in both the maintenance of nicotine addiction and the capacity for successful cessation. Early proof-of-concept studies demonstrated that high-frequency repetitive TMS applied to the left dorsolateral prefrontal cortex could acutely reduce cigarette craving and consumption [23,24]. These initial findings generated significant research interest, and many randomized controlled trials were carried out on different forms of TMS techniques, targets, and parameters. The evidence from all these trials has now resulted in regulatory approvals, with Zangen et al. showing in a pivotal multicenter trial that deep TMS with use of the H4 coil targeting bilateral prefrontal cortex and insula reached 28.4% four-week continuous quit rates versus 11.7% with sham stimulation [25]. This landmark trial provided the evidentiary basis for the United States Food and Drug Administration clearing the BrainsWay deep TMS system with H4 coil for short-term smoking cessation in 2020 [26], followed by Health Canada’s authorization in 2022 [27]. It is important to note, however, that the pivotal trial had a relatively short follow-up window and a high placebo response in the sham arm, which tempers the strength of the regulatory milestone as evidence of long-term clinical efficacy. These regulatory milestones together signal important validation of TMS as a clinically viable intervention for nicotine addiction.

Despite the increasing clinical use of TMS for smoking cessation and the increasing evidence of its effectiveness, there are still many essential knowledge gaps regarding the neurobiological mechanisms of therapeutic effect. However, it is generally presumed that TMS exerts benefits through modulation of functional connectivity within executive control and salience networks, and direct effects of cortical TMS on subcortical dopaminergic neurotransmission remain relatively unexplored [26]. Recent neuroimaging studies have started to detail some of these mechanisms: using high-frequency repetitive TMS targeted to the left dorsolateral prefrontal cortex, Wang et al. showed that the approach is associated with changes in reward circuitry functional connectivity in nicotine-dependent individuals, with changes in connectivity patterns correlating with reductions in craving severity; though these are correlational findings that do not establish a causal mechanism [28]. It is not known, however, the extent to which such neural changes predict long-term abstinence outcomes, or the degree to which changes vary across different TMS protocols. Moreover, significant variability has been observed regarding different treatment outcomes across clinical trials. Moderator effects suggest that younger individuals, those with higher educational attainment, and those with lower preceding histories of smoking tend to show superior outcomes after deep TMS therapy [29]. However, it is not understood at present at what neurobiological level these different outcomes occur, or whether patterns can be used to direct therapeutic selection.

The current narrative review attempts to bridge this knowledge gap by synthesizing existing literature on neurobiological mechanisms underlying the efficacy of TMS approaches on smoking cessation, with a focus on modulating craving and nicotine dependence through the restoration of neural circuits. We aim to develop an extensive framework on how different TMS modalities influence various neural circuits involved in nicotine addiction, and hence, the resulting effect on dependence, craving, and addiction, and which factors moderate these relationships. This synthesis, from various research by functional magnetic resonance imaging, positron emission tomography, and clinical studies, is aimed at improving the application and understanding of TMS for smoking cessation, ultimately contributing to the improvement of neuroscience-based interventions for nicotine addiction.

## 2. Materials and Methods

An extensive literature search was conducted using five major online databases, including PubMed/MEDLINE, Embase, Web of Science, PsycINFO, and Cochrane Central Register of Controlled Trials, covering publications from January 2015 to January 2026. Three conceptual domains were combined using Boolean operators: Intervention terms included “transcranial magnetic stimulation,” “rTMS,” “deep transcranial magnetic stimulation,” and “theta burst stimulation.” Condition-related terms included “smoking,” “nicotine dependence,” “tobacco use,” and “smoking cessation.” Mechanism/outcome-related terms included “craving,” “functional connectivity,” “neuroimaging,” “reward circuit,” “prefrontal cortex,” and “neural plasticity.” Reference snowballing was performed on retrieved articles to identify additional relevant studies not captured in the initial database searches.

The full Boolean search string used across databases was as follows: (“transcranial magnetic stimulation” OR “rTMS” OR “deep transcranial magnetic stimulation” OR “theta burst stimulation”) AND (“smoking” OR “nicotine dependence” OR “tobacco use” OR “smoking cessation”) AND (“craving” OR “functional connectivity” OR “neuroimaging” OR “reward circuit” OR “prefrontal cortex” OR “neural plasticity”). This string was adapted to the syntax of each database as needed. The complete database-specific search strings are provided in Supplementary Table S1.

Following the initial search, duplicate records were removed. Titles and abstracts were then screened against the eligibility criteria by two independent reviewers, with disagreements resolved by consensus. Full texts of potentially eligible studies were retrieved and assessed. A total of 34 studies met all inclusion criteria and were incorporated into this narrative synthesis. Although a PRISMA flow diagram was not pre-registered, the selection process followed PRISMA reporting principles to the extent applicable to a narrative review. The primary reason a formal systematic review methodology was not adopted is the substantial heterogeneity across TMS protocols, outcome measures, and study designs, which precluded a meaningful meta-analytic synthesis independent of the existing meta-analyses already incorporated.

Studies were selected based on predefined eligibility criteria. Included investigations enrolled adults aged 18 years or older with nicotine dependence or tobacco use disorder. Any TMS protocol, including high-frequency repetitive TMS, low-frequency repetitive TMS, deep TMS, and theta burst stimulation, directed to any region of the cortex, was eligible. Comparators included sham stimulation, standard treatments for cessation, or no treatment. Results needed to relate either to craving scores, measures of nicotine dependence, abstinence rates, or neural imaging. Designs of interest included randomized controlled trials, non-randomized studies, single-arm trials, and neuroimaging studies. Mechanistic neuroimaging studies (fMRI, PET, DTI) that did not include formal cessation outcomes but provided direct evidence of TMS effects on addiction-relevant neural circuits were also included, as they are integral to addressing the mechanistic aims of the review. Non-TMS neuromodulation studies (e.g., tDCS) and pharmacological challenge paradigms were included selectively and only where they provided mechanistic insight into circuits targeted by TMS; these are clearly labeled as non-TMS studies throughout. Non-English language reports, case reports with fewer than ten participants, and articles with no original data were not included.

Data extraction captured study characteristics, including author, year, design, and sample size. Participant information included demographics, baseline information about smoking, and comorbid conditions. Intervention characteristics included modality, frequency, and intensity of TMS, including session count, site, and type. Outcome data extracted included craving, dependence, abstinence, and neuroimaging, including connectivity and site changes. Quality was appraised narratively with reference to key domains of internal validity (randomization, blinding, sham credibility, biochemical verification of abstinence, follow-up duration, and sample size), although no formal risk-of-bias tool was applied, consistent with the narrative design of this review. Where quality considerations bear on the interpretation of findings, these are noted within each thematic section.

Evidence synthesis used thematic narrative integration instead of quantitative integration since there are considerable heterogeneities between the protocols employed, outcome measures used, and follow-up periods. To provide a transparent framework for interpreting evidence, findings are organized by a three-tier hierarchy: (1) evidence from multi-site RCTs and meta-analyses; (2) evidence from smaller single-site RCTs and controlled clinical trials; and (3) mechanistic and neuroimaging findings from small or single-session studies. Readers should weigh conclusions accordingly. Findings were synthesized to include several domains: neurobiological underpinnings of addiction, mechanisms of TMS, effects of craving, outcomes of drug dependence, modification of neural circuits, and moderators of treatment response. This approach was effective in organizing diverse studies, their similarities, inconsistencies, and gaps.

## 3. Results

### 3.1. Neurobiological Foundations of Nicotine Dependence

#### Reward Circuitry Dysfunction

Nicotine addiction is a result of alterations in the brain reward system, which are mediated by the nicotinic acetylcholine receptors [30,31]. The ligand-gated ion channels control the release of neurotransmitters, and the high-affinity α4β2-containing nicotinic acetylcholine receptors are densely located on the ventral tegmental area dopaminergic neurons [30]. This is done by activating presynaptic acetylcholine receptors, which enhance dopamine release in the nucleus accumbens, leading to reward system adaptations in the mesolimbic system [31]. The β3, α6, and α5 subunits combine to form various acetylcholine receptor subtypes that affect dopamine release, while the low-affinity α3/β4 acetylcholine receptor subtype is found in brain areas associated with aversion, such as the medial habenula and interpeduncular nucleus [30].

Chronic exposure results in neuroadaptive changes, which alter drug-related and natural reward responses [31,32]. Nicotine dependence results in increased ventral striatum and orbitofrontal cortex responses to smoking-related cues and decreased responses to non-drug reward cues [31]. Addicott et al. [32] observed that tobacco withdrawal significantly reduced the activation in the supplementary motor area and the ventromedial prefrontal cortex in the anticipation of reward. The reduction in activation was inversely related to the Fagerström Nicotine Dependence Score. Conversely, nicotine in non-smokers enhances nucleus accumbens differentiation between rewarded and non-rewarded outcomes, suggesting that the feedback salience is high [32]. In addition, it modulates GABAergic, serotonergic, and noradrenaline systems [32]. Nicotine also inhibits the breakdown of dopamine via monoamine oxidase [32]. In the case of schizophrenia, nicotine can compensate for neurochemical deficits via interactions with dopamine, glutamate, and GABA [33].

### 3.2. Executive Control Deficits

Nicotine dependence has been found to significantly impair prefrontal cortex-mediated executive functions, such as inhibitory control [34,35]. Inhibitory control has been shown to be strongly related to nicotine dependence severity among executive components [34]. Compared to smokers with moderate levels of dependence, smokers in the higher dependence group have shown significant impairments in inhibitory control (Stroop and Hayling tests) and updating processes (measured via n-back tasks) [34]. The inhibitory capacity emerged as the strongest predictor of the severity of nicotine dependence, even superior to craving and demographic factors [34].

The neuroanatomical substrates involve not only isolated prefrontal areas but also distributed networks, with cortico-striatal dopaminergic systems playing critical roles through dorsal striatal projections and reciprocal connections with the prefrontal areas [35]. Neural changes following chronic nicotine use are wide-ranging, affecting both the expression of the receptors and the functional connectivity in the brain [35]. New developments in the fields of pharmacogenetics and optogenetics enable the precise dissection of the roles of particular types of receptors in cognitive deficits [35]. Metacognitive deficits represent an additional dimension, with smokers demonstrating reduced metacognitive accuracy compared to non-smokers, manifesting as systematic overestimation of subjective value placed on delayed rewards [36]. This impairs the ability to detect preference reversal risks in voluntary restrictions, which makes it difficult to detect vulnerability to immediate temptations [36].

### 3.3. Salience Network Alterations

The salience network, comprising the anterior insula and the dorsal anterior cingulate cortex, is responsible for the detection of behaviorally relevant stimuli [37]. In the context of nicotine dependence, it is associated with abnormal functioning and drug cue sensitivity as well as interoceptive abnormalities. In sated smokers compared with controls, resting-state studies revealed greater interhemispheric coupling, especially frontally, which positively correlated with daily cigarette use [37,38]. Conversely, diffusion tensor imaging demonstrated negative correlations between genu corpus callosum white matter streamlines and cigarettes smoked daily, suggesting structural connectivity alterations despite functional hyperconnectivity [38].

The negative effect of nicotine withdrawal, which is channeled through the dysregulation of the salience network and stress system interaction, results in feelings of anxiety, irritability, and decreased concentration through neuroadaptations within the system itself [39]. Social defeat stress enhances the negative effect of nicotine withdrawal by disrupting neuropeptide signaling in nicotinic receptor systems [39]. Recent circuit neuroscience conceptualizes addiction as a disorder of neural circuit function, with functional magnetic resonance imaging providing information flow details at behaviorally relevant resolution [37].

## 4. TMS Mechanisms in Smoking Cessation

### 4.1. Neurophysiological Principles

Repetitive transcranial magnetic stimulation (rTMS) functions as a non-invasive, drug-free, neural-circuit-based therapeutic tool recently cleared by the United States Food and Drug Administration for smoking cessation treatment [26]. The therapeutic technique focuses on executive control, default mode, and salience networks by neuromodulation of the bilateral dorsolateral and medial prefrontal cortices, cingulate, and insula [26]. High-frequency stimulation increases excitability, and low-frequency stimulation decreases it [40]. It also depends on the target areas, frequencies, intensity, and time, as well as the number of sessions and the patient, including age, medication, and symptoms [40]. Chronic smoking affects the prefrontal and meso-cortical limbic systems, making the frontal areas suitable for neuromodulation [41]. Sequential application of 20 Hz stimulation to the left dorsolateral prefrontal cortex and superior medial frontal cortex demonstrated significant smoking craving reduction and resting brain activity reduction measured by cerebral blood flow and brain entropy after ten treatment days [41]. While the presumed mechanism involves modulating functional connectivity circuits engaged in executive control and salience processing of drug-related cues, any claim that TMS directly modulates subcortical dopaminergic transmission remains hypothetical; this pathway has not been directly confirmed in the smoking cessation literature and would require multimodal PET-fMRI investigation to establish [26]. TMS enhances neuronal plasticity, especially long-term potentiation/depression, and controls brain-derived neurotrophic factor gene expression [40]. Repetitive TMS has the greatest promise in neuromodulation techniques, and strong research has shown it to be effective in reducing cravings for substances [42].

### 4.2. Target Brain Regions

The dorsolateral prefrontal cortex (DLPFC) is the most investigated target for rTMS-based smoking cessation therapy, with the ventromedial prefrontal cortex (vmPFC), anterior cingulate cortex (ACC), and insula being investigated as increasingly important target areas. The insula deserves particular mention: lesion studies have demonstrated that insular damage can precipitate spontaneous smoking cessation, and deep TMS with H-coils capable of reaching the insula is the basis of the FDA-cleared BrainsWay protocol [25]. In the study by Aronson Fischell et al. [43], a session of anodal left DLPFC and cathodal rTMS of the right vmPFC significantly increased the deactivation of default mode network nodes during working memory and ACC activity during error monitoring. Smokers exhibited greater stimulation-induced default mode network deactivation while sated compared to withdrawn and greater ACC activity during error monitoring compared to non-smokers [43]. This study used tDCS, not TMS, and is included here as a mechanistic probe of the same prefrontal circuits targeted by rTMS; it does not constitute direct evidence for rTMS efficacy. Withdrawal negatively impacts inhibitory control, with abstinence-induced decreases in activation within occipital and parietal cortex during sustained inhibition associated with changes in bilateral insula activation [44]. Active stimulation applied to the dorsolateral prefrontal cortex decreased activity in the contralateral medial orbitofrontal cortex and ipsilateral nucleus accumbens compared to sham conditions among nicotine-dependent smokers [45]. Treatment characteristics across these investigations are summarized in Table 1. Cognitive circuit dysregulation associated with nicotine withdrawal may be modifiable through dual-target approaches, with potentially stronger effects as a complement to existing therapies such as nicotine replacement due to possible enhanced plasticity in the sated state [43].

### 4.3. Protocol Variations

Significant heterogeneity exists in the protocols of smoking cessation using rTMS, particularly in the frequency, intensity, treatment duration, and number of sessions. This heterogeneity substantially limits direct cross-study comparison and is a key reason why definitive conclusions about optimal parameters remain elusive. A randomized factorial study using 900 pulses at 20 Hz to the left DLPFC, varying in duration (8, 12, 16 days) and daily sessions (1 or 2 sessions), showed a large effect size for duration and a medium effect size for intensity in the latency to relapse. Ten daily MRI-guided sessions over two weeks to the left dorsolateral prefrontal cortex paired with craving cues significantly reduced biochemically confirmed cigarette consumption compared to sham during treatment and at one-month follow-up, with active stimulation participants more likely to quit by their target quit date [46]. A more lateral coil location was associated with greater success in quitting across the active treatment sample [46]. High-frequency protocols demonstrated efficacy in improving nicotine-related dependence by modulating local neural synchronization in the prefrontal cortex and angular gyrus, with post-treatment regional homogeneity values in the left middle frontal gyrus negatively correlated with post-treatment craving scores [47]. Treatment characteristics across protocol variation studies are presented in Table 2. Accelerated intermittent theta burst stimulation (four sessions per day for five consecutive days) over the left dorsolateral prefrontal cortex reduced cigarette consumption, nicotine dependence, craving, and perceived stress equally across active and sham conditions [48]. This null result (where sham performed equivalently to active stimulation) is one of the most methodologically significant findings in this literature. The authors attribute this partly to the use of advanced sham-coil technology, which improves placebo credibility and may amplify non-specific expectancy effects. This study is an important reminder that effect sizes in open or weakly blinded trials are likely inflated, and that placebo response rates in TMS trials for addiction can be substantial. It should not be dismissed as simply one trial among many; rather, it highlights the urgent need for rigorous sham-controlled designs. Continuous theta burst stimulation to the right inferior frontal gyrus improved inhibitory control task performance compared to intermittent theta burst stimulation, with both conditions reducing cigarette craving and smoking compared to baseline [49]. Continuous theta burst stimulation-induced improvement in inhibitory control performance was associated with reduced craving, which was associated with reduced smoking [49].

Across these studies, the treatment sessions range from one session to several weeks, thresholds vary from 80% to 120%, and the frequencies used are 10–20 Hz, including theta burst, with 900–3000 pulses per session. These variations pose challenges in the synthesis of evidence and the standardization of recommendations. Extended high-frequency stimulation of the left DLPFC appears most effective in the reduction of smoking and craving. However, theta burst stimulation and other cortical targets should be the focus of further trials of longer-term follow-up to determine the optimal characteristics of responders.

## 5. Clinical and Neurobiological Outcomes of TMS in Smoking Cessation

### 5.1. Effects on Craving

One of the central aspects of nicotine addiction is craving, which is also a major target of transcranial magnetic stimulation. Table 3 presents findings organized by evidential weight: the multicenter RCT and meta-analysis first, followed by smaller controlled trials, then single-session mechanistic studies. The most substantial evidence is that of Zangen et al. [25], where a multicenter trial with 262 chronic smokers showed that bilateral stimulation of the lateral prefrontal and insular cortices after cue-induced procedures reduced craving more in the active than the sham group as early as two weeks into the study. Daily MRI-guided sessions over two weeks paired with video smoking cues significantly reduced mean craving throughout treatments and at one-month follow-up among treatment-seeking nicotine-dependent smokers [46]. Active participants receiving 10 Hz stimulation with 3000 pulses per session demonstrated sustained craving reductions extending beyond acute treatment periods [46].

The measurement tools used were the Tobacco Questionnaire for Smoking Urges, the Visual Analog Scale, and several craving subscales. Table 3 presents the treatment characteristics and craving results from the key studies. A meta-analysis of 17 RCTs involving 859 participants found a significant effect in the Tobacco Questionnaire of Smoking Urges, where the mean difference was −10.89 in favor of active stimulation [51]. However, no significant effects were found for the Visual Analog Scale, suggesting a problem of measurement sensitivity or differential response to acute and sustained craving states [51]. These discrepant findings across craving instruments highlight the importance of standardizing outcome measurement: the field currently lacks consensus on whether brief acute VAS ratings or multi-item questionnaires like the TQSU better capture clinically meaningful craving reductions. In males, active treatment for 15 sessions over 3 weeks was found to have a greater effect on craving than sham treatment, and this was sustained by a decrease in urinary cotinine [52].

Cue-reactivity modulation represents an additional mechanism through which stimulation may exert therapeutic effects. High-frequency protocols targeting the left dorsolateral prefrontal cortex reduced nicotine dependence and alleviated withdrawal symptoms, with functional connectivity changes in reward circuitry associated with reductions in smoking scale scores [28]. The right nucleus accumbens showed increased functional connectivity with multiple visual processing regions following treatment, a finding consistent with stimulation modulating neural representation and salience of smoking-related cues [28]. These are correlational neuroimaging findings from a small sample (*n* = 17) and should not be interpreted as establishing a causal mechanism. Single-session stimulation targeting different cortical nodes revealed that superior frontal gyrus stimulation significantly reduced craving versus control conditions, with larger effects in men compared to women [53]. Theta burst protocols targeting the right inferior frontal gyrus reduced cigarette craving and smoking compared to baseline conditions, with continuous theta burst stimulation-induced improvement in inhibitory control associated with reduced craving and subsequent smoking reduction [49]. Significantly, accelerated intermittent theta burst stimulation reduced craving equally across active and sham conditions, indicating substantial placebo effects potentially amplified when utilizing advanced placebo coil technology [48].

### 5.2. Effects on Nicotine Dependence

Nicotine dependence represents a core construct in tobacco use disorder, quantified primarily through the Fagerström Test for Nicotine Dependence and accompanied by objective measures of cigarette consumption and biochemically verified abstinence. Repetitive TMS of the left DLPFC over 10 sessions reduced FTND scores compared with baseline, which eased withdrawal symptoms and reduced cigarette consumption [54]. Four subjects stopped smoking after treatment, and there was a strong positive correlation with changes in cigarette use and axial diffusivity of right nucleus accumbens fibers [54]. Two-week treatment reduced craving in both active and sham groups, with active rTMS increasing regional homogeneity in left orbital inferior frontal gyrus, left middle frontal gyrus, and right angular gyrus, compared with a reduction in sham subjects [47]. Left middle frontal gyrus regional homogeneity after treatment negatively correlated with craving, and this neuroimaging measure may predict treatment response [47]. A multicenter trial of 262 chronic smokers found bilateral rTMS of lateral prefrontal and insular cortices resulted in quit rates of 19.4% vs. 8.7% at four weeks in an intent-to-treat analysis, and 28.0% vs. 11.7% in completers, active vs. sham, respectively [25]. These quit rates were assessed via self-report and were not biochemically verified in all participants, which is a limitation of the trial. The relatively short follow-up (end of treatment through week 18) also limits conclusions about durability. The sham response rate of 8.7–11.7% is notable and underscores the importance of placebo effects in this population. Cigarette consumption and craving reductions were significantly greater in active versus sham groups as early as two weeks into treatment [25]. Treatment outcomes across investigations examining nicotine dependence are presented in Table 4. Functional connectivity-guided rTMS to PFC areas, based on common neural circuits for schizophrenia and nicotine addiction, decreased FTND scores after 15 and 20 sessions vs. sham rTMS in schizophrenia patients [55]. Greater increases in resting-state functional connectivity predicted larger reductions in cigarettes per day among patients receiving active treatment [55]. The finding of enhanced rTMS efficacy in schizophrenia patients is clinically interesting but requires careful interpretation. Antipsychotic medications (particularly dopamine D2 receptor antagonists) substantially alter the dopaminergic milieu in ways that could interact with both the addictive mechanisms of nicotine and the neuromodulatory effects of rTMS. Future studies in this population should control for antipsychotic type, dose, and duration as potential effect modifiers.

An observational sub-analysis of the randomized trial on deep TMS, in which cue-restricted smoking was added as an aide, demonstrated that further consumption reductions occurred in 40%, and 16.5% had verified cessation by week 12 [56]. The cue-restricted form of smoking, restricted to standing and wall-facing in sensory-deprived environments, was linked with meaningful reductions and verified cessation, even in the absence of significant treatment effects in the main trial on deep TMS [56]. Active rTMS, as compared with control, significantly reduced daily cigarette consumption by 6.72 cigarettes and decreased the FTND by 2.57 points, with cessation outcomes favoring rTMS (OR 2.77) in the meta-analysis of 15 RCTs involving 967 participants [57]. Stimulation at 10 Hz, as opposed to 0 Hz, produced better outcomes across all measures, and 20+ sessions produced better outcomes in cessation, consumption, and nicotine dependence [57]. In the patient group with schizophrenia, rTMS had greater efficacy in reducing smoking and nicotine dependence, suggesting potential population-specific treatment responsiveness [57]. It is important to note that the conclusions regarding 10 Hz superiority and the ≥20-session threshold derive from the existing meta-analyses [51,57] rather than from systematic re-analysis of the included studies within this review; as such, these should be treated as the best available but not definitive parameters, given heterogeneity in the underlying trials.

### 5.3. Neural Circuit Restoration

Neuroimaging studies demonstrate the effect of rTMS on the neural circuitry of smoking cessation. fMRI studies demonstrate acute and enduring changes in large-scale networks. rTMS at 20 Hz, targeting the left DLPFC and superior medial frontal cortex, for 10 days decreased resting brain activity, as indexed by cerebral blood flow and brain entropy. About 90% of the completers were abstinent during the 25-day follow-up period [41]. The observed reductions in cerebral blood flow and brain entropy measurements revealed that high-frequency rTMS produces measurable alterations in cortical activity patterns [41]. Salience network connectivity emerged as a critical predictor of treatment response, with higher salience connectivity during task-based fMRI and lower reward connectivity during resting-state fMRI predicting better cessation outcomes [47]. The opposite directions of association depending on imaging paradigm (task vs. rest) warrant caution and suggest that connectivity biomarkers may be context-dependent; this finding needs replication in larger samples before it can guide clinical selection.

Brain connectivity network analysis reflects alterations in functional and structural connectivity networks comprising adjacent and separated regions related to stimulation sites, demonstrating patterns consistent with intrinsic functional integration and neuroplasticity [58]. Repetitive TMS exerts stimulating effects on neuronal plasticity processes, particularly long-term potentiation and depression mechanisms, alongside regulation of gene expression associated with brain-derived neurotrophic factor synthesis [40]. Eight sessions of 20 Hz rTMS over the left DLPFC, in addition to self-help, reduced delay discounting and reduced relapse threefold, increasing abstinence rates from 15.4% (sham rTMS) to 50% (active rTMS) [59]. MRI-based brain connectivity can help identify neural signatures of treatment response, which will help understand rTMS mechanisms and inform smoking cessation plans [58]. These neuroimaging findings, while mechanistically informative, collectively derive from studies with small samples and should be understood as generating hypotheses for future confirmatory investigation, not as establishing that TMS restores addiction-disrupted circuits in a proven or irreversible manner.

## 6. Moderators of Treatment Response

Treatment response to TMS in smoking cessation varies substantially based on demographic characteristics, baseline smoking patterns, and stimulation protocol parameters. Demographics play a role in deep TMS treatment outcomes. Younger individuals, higher education level, and fewer smoking habits were associated with a good outcome [29]. Those under 40 benefited with 32% quitting compared with 18% of those over 40, while college graduates benefited with 35% quitting compared with 21% of those with less education [29]. There were also differences by gender, as the superior frontal gyrus was found to be more active in men compared with women in response to stimulation for craving reduction [53].

The baseline smoking traits were found to be important, as there was a correlation of reduced nicotine dependency with increased rTMS benefit [29]. The resting-state DLPFC–nucleus accumbens connectivity was predictive of rTMS outcome, as each person’s circuit architecture is different [45]. Population-specific effects were observed in schizophrenia, where rTMS appeared to be particularly effective in reducing smoking and nicotine dependence [55,57]. As noted in Section 5.2, the confounding influence of antipsychotic medication on dopaminergic systems means that these findings cannot be straightforwardly attributed to rTMS effects on the addiction circuit alone and should be interpreted with appropriate caution.

The effectiveness of the treatment protocol also depends on the specific parameters, with frequency, numbers, and duration showing different results. The meta-analysis by Shang et al. [57] showed that stimulation at 10 Hz was better than stimulation at 0 Hz in terms of craving, consumption, and dependence. Longer treatment sessions (more than 20 sessions) enhance cessation, reduce consumption, and decrease nicotine dependency compared to shorter sessions [57]. The probability of increasing abstinence by 7–8 times for each additional period of treatment, as well as doubling the probability of abstinence with increased session intensity, was found, with strong effects of duration and moderate effects of intensity on relapse latency [50]. More lateral coils were associated with higher quit rates, which means precise anatomical targeting is important [46]. A critical limitation of the moderator literature is that most analyses are exploratory and post hoc, derived from secondary analyses of trials not specifically powered to detect moderator effects. Prospective studies with pre-specified moderator hypotheses, adequate sample stratification by age, sex, education, and baseline dependence, and integration of neuroimaging biomarkers are needed before personalized treatment selection based on these variables can be recommended in clinical practice.

## 7. Discussion

Tobacco smoking is a formidable public health challenge despite the availability of evidence-based cessation interventions, with relapse rates exceeding 70% within the first year following quit attempts. Neuronal advances have shown the neurobiological mechanisms underlying nicotine dependence, emphasizing transcranial magnetic stimulation’s potential for neuromodulation of addicted neural circuits. Currently, the results of both pharmacological and behavioral treatments are poor in the long term, with <30% achieving abstinence after a year [7]. Repetitive TMS is a non-invasive technique that has shown potential in treating nicotine dependence, particularly in treatment-resistant individuals, by influencing the brain networks associated with the addiction [60,61].

Mechanistically, rTMS targets various neurobiological levels, with effects on reward system activity that are thought to involve modulation of dopaminergic, GABAergic, and glutamatergic transmission, although direct evidence for these subcortical effects in the smoking cessation context remains limited and largely inferential [26]. Repetitive TMS also increases neuroplasticity through long-term potentiation/depression and genes involved in brain-derived neurotrophic factor synthesis [40]. In terms of networks, rTMS is associated with changes in the default mode network and normalizes the connectivity of salience/executive control networks, thus partially counteracting imbalances in the networks resulting from chronic nicotine exposure [43,47]. In terms of behavior, rTMS has advantages that include better inhibitory control, reduced preference for immediate rewards, and enhanced executive functions that include working memory and cognitive flexibility, thus showing promise for treating cognitive deficits in addiction [34,59]. These behavioral observations align with, but do not by themselves confirm, a circuit-restoration model of TMS action; much of the underlying mechanistic evidence is correlational and derived from small neuroimaging studies. Interventions directly address circuit pathologies of addiction, namely, prefrontal cortex hypofrontality, mesolimbic system hyperactivity, and maladaptive synaptic remodeling, as described in Koob et al. [62].

Repetitive TMS, using magnetic pulses, decreases nicotine dependence, craving, and smoking. The pivotal multicenter trial by Zangen et al. [25] proved that bilateral stimulation of the lateral prefrontal and insular cortices resulted in 19.4% continuous quit rates compared to 8.7% with sham stimulation, providing the evidentiary foundation for FDA clearance. In a meta-analysis of 967 smokers, significant reduction in the number of cigarettes smoked per day, FTND scores, and increased quit rates (OR 2.77) were found in the active treatment group [57]. The DLPFC has been studied most extensively for executive/top-down reward control [63]. However, new findings also support other regions; for example, the superior frontal gyrus reduced cravings in a sex-specific manner (males vs. females) [53], as did a key node of the hyperdirect pathway, namely, the right inferior frontal gyrus, involved in inhibitory control [49].

Deep TMS using H-coil designs offers a broader cortical stimulation field and a deeper magnetic field penetration compared with standard figure-eight coils, which might be advantageous in treating complex networks involved in addiction, such as the insular cortex [25]. The insula, as a network for interoception and conscious craving, appears to be a promising target, as lesion studies have demonstrated that damage to this region can result in spontaneous smoking cessation [64]. The potential advantages of theta burst stimulation protocols, with their short treatment sessions and robust neuroplastic changes, are promising, even if evidence for superiority over conventional high-frequency protocols is equivocal. Crucially, the Mikellides et al. [48] accelerated iTBS trial (which found no difference between active and sham stimulation) should be centrally integrated into the interpretation of this literature. This is not simply a null result to be balanced against positive trials; it is a methodological signal that placebo effects in TMS addiction trials may be substantially inflated when sham coil technology is highly credible. The field must contend with this possibility in designing and interpreting all future trials. Expectancy, therapeutic alliance, and contextual cue exposure likely contribute meaningfully to treatment outcomes across active and sham conditions.

Neuroimaging studies have yielded important mechanistic findings, indicating rTMS modulates functional connectivity within and between large-scale brain networks involved in addiction neurocircuitry. Salience network connectivity emerged as the strongest predictor of treatment response across both task-based and resting-state paradigms, though connectivity predicted outcomes in opposite directions depending on imaging context [47]. An increase in functional connectivity between the nucleus accumbens and visual processing areas was related to craving reductions, indicating that rTMS normalizes the representation and motivational significance of smoking cues [28]. These changes in circuitry are examples of intrinsic functional integration and neuroplasticity, indicating that cortical stimulation can have cascading effects on subcortical and distributed systems [58]. However, these findings derive from small samples (*n* = 17 in the Wang et al. [28] study) and the absence of follow-up neuroimaging data means it cannot be confirmed whether connectivity changes are durable or mechanistically responsible for clinical outcomes.

Treatment response demonstrates substantial heterogeneity moderated by demographic characteristics, baseline smoking patterns, and protocol parameters. Young age, educational level, and baseline dependence severity are positive predictors, suggesting that the earlier the treatment, the better the outcome, and cognitive resources facilitate treatment outcome [29]. However, as noted, these moderator findings are largely post hoc and should be considered hypothesis-generating. Optimizing techniques are continuously evolving, and meta-analyses have shown the efficacy of 10 Hz, more than 20 sessions, and precise targeting with the help of neuroimaging techniques [46,57]. Population-specific responsiveness of schizophrenia indicates the possibility of tailored treatment [55], though the confounding role of antipsychotic medication on dopaminergic systems must be controlled in future studies before conclusions about the neurobiological basis of this effect can be drawn.

Despite the rising body of evidence demonstrating the efficacy of rTMS treatment, existing limitations with different stimulation parameters, target localization, treatment schedules, and outcome measures are impeding cross-study synthesis and standard protocol development [51]. The neurobiological mechanisms by which cortical stimulation influences subcortical dopamine systems need to be further explored by multimodal neuroimaging techniques, which include positron emission tomography [65]. A specific and underappreciated gap is the inconsistency in abstinence measurement: some studies use biochemically verified continuous abstinence, others use self-reported point prevalence, and follow-up windows range from immediately post-treatment to 18 weeks. Future studies should report outcomes using standardized definitions: biochemically verified, continuous abstinence at 6 months and 12 months post-treatment, consistent with best practice in smoking cessation research. Future research should also prioritize adequately powered randomized controlled trials with harmonized protocols, extended follow-up assessments, integration of predictive biomarkers for treatment selection, and investigation of combination approaches pairing rTMS with pharmacotherapy or behavioral interventions to optimize cessation outcomes and address the persistent public health burden of tobacco smoking.

## Figures and Tables

**Table 1:** Treatment characteristics and outcomes across investigations of brain region-specific TMS for smoking cessation.

Author and Year	N	Technique	Target	Stimulation Parameters	Treatment Number	Outcomes
Aronson Fischell et al., 2020 [43]	15 smokers, 28 non-smokers (non-TMS mechanistic study)	tDCS	L-DLPFC (anodal)/R-vmPFC (cathodal)	2 mA, 20 min	1 session	Enhanced DMN deactivation during working memory; strengthened ACC activity during error monitoring
Sweitzer et al., 2018 [44]	37 (17 ADHD, 20 non-ADHD) (pharmacological probe)	Methylphenidate challenge during fMRI	Occipital/parietal cortex, bilateral insula	40 mg MPH during 24 h abstinence	1 session	Abstinence decreased occipital/parietal activation; MPH improved performance and increased sustained inhibitory control activation
Li et al., 2017 [45]	11 non-treatment seeking nicotine-dependent cigarette smokers	HF-rTMS	L-DLPFC	10 Hz, 5 s on/10 s off, 100% MT, 3000 pulses	1 session	Decreased mOFC and ipsilateral NAc activity compared to sham

Note: Aronson Fischell et al. [43] used tDCS (not TMS), and their study is included as a mechanistic comparator; Sweitzer et al. [44] used a pharmacological fMRI paradigm. These are clearly distinguished from TMS-only studies.

**Table 2:** Treatment characteristics and outcomes for different TMS protocol approaches in smoking cessation.

Author and Year	N	Technique	Target	Stimulation Parameters	Treatment Number	Duration	Outcomes
Shevorykin et al., 2022 [50]	23	HF-rTMS	L-DLPFC	20 Hz, 900 pulses, 1 or 2 sessions/day	8, 12, or 16 days	8–16 days	Increasing duration increased abstinence odds 7–8 fold; increasing intensity doubled odds
Li et al., 2020 [46]	42	HF-rTMS	L-DLPFC (MRI-guided)	10 Hz, 3000 pulses/session with video cues	10 daily sessions	2 weeks	Active: fewer cigarettes during treatment and follow-up; 23.81% quit vs. 0% sham
Li et al., 2025 [47]	31 (19 rTMS, 12 sham)	HF-rTMS	L-DLPFC	10 Hz	10 sessions	2 Weeks	Increased ReHo in L-MFG; ReHo negatively correlated with craving
Mikellides et al., 2022 [48]	89	aiTBS	L-DLPFC	iTBS, 4 sessions/day	20 Sessions	5 Days	Active = Sham: no difference. Large placebo effect; methodologically critical null result.
Upton et al., 2023 [49]	37	iTBS and cTBS	R-IFG	Theta burst patterns, 80% MT|1 session each	1 Session each	Single session	cTBS improved IC vs. iTBS; both reduced craving and smoking

**Table 3:** Craving assessment approaches and treatment outcomes in TMS trials for smoking cessation.Ordered by evidential weight (multicenter RCT/meta-analysis → smaller RCTs → single-session mechanistic studies).

Author and Year	N	Target Region	Session Number	Craving Instrument	Primary Craving Outcome	Follow-Up Duration
**Tier 1 (Multicenter RCT/Meta-analysis)**						
Zangen et al., 2021 [25]	262	Bilateral lateral PFC and insula	21 sessions	Post-cue craving	Greater reduction active vs. sham from week 2	18 weeks
Ismail et al., 2025 [51]	859 (meta-analysis)	Various	Various	TQSU; VAS	TQSU: MD = −10.89 (*p* < 0.00001); VAS: nonsignificant	Various
**Tier 2 (Smaller RCTs)**						
Li et al., 2020 [46]	42	L-DLPFC (MRI-guided)	10 daily	Mean craving score	Significant reduction (*p* < 0.001)	1 month
Mousa et al., 2025 [52]	40	L-DLPFC	15 sessions	Craving scale	Significant reduction (*p* = 0.007) vs. sham	Follow-up period
Mikellides et al., 2022 [48]	89	L-DLPFC	20 sessions	General craving	Active and sham equally reduced; lasted ≥1 week	1 week
**Tier 3 (Single-session/Mechanistic)**						
Wang et al., 2024 [28]	17	L-DLPFC	10 sessions	Smoking craving scales	Reduced; correlated with NAc connectivity	Not specified
Petersen et al., 2025 [53]	72	SFG, dlPFC, PPC, v5	1 session	Self-report craving	SFG reduced craving vs. control; men > women	Immediate
Upton et al., 2023 [49]	37	R-IFG	1 session each	Cigarette craving	Both iTBS and cTBS reduced vs. baseline	Immediate

**Table 4:** Nicotine dependence outcomes across transcranial magnetic stimulation intervention studies.

Author and Year	N	Target Region	Sessions	FTND/Dependence Outcome	Consumption Reduction	Abstinence Rate	Key Findings
Chen et al., 2025 [54]	18	L-DLPFC	10	FTND significantly decreased	Significant reduction; 4 achieved cessation	22.2% cessation	Changes correlated with NAc fiber alterations
Li et al., 2025 [47]	31	L-DLPFC	10	Craving reduced both groups	Not specified	Not specified	ReHo in L-MFG negatively correlated with craving
Zangen et al., 2021 [25]	262	Bilateral lateral PFC and insula	21	Not primary outcome	Greater reduction vs. sham	Active: 19.4%; Sham: 8.7% (ITT) self-report, limited verification	Completers: 28.0% vs. 11.7%
Du et al., 2021 [55]	30	PFC (connectivity-guided)	20	FTND reduced after 15, 20 sessions (*p* < 0.05)	CPD reduction not significant vs. sham	Not specified	rsFC increase predicted CPD reduction
Scholz et al., 2025 [56]	85	Deep TMS	Combined with CRS	Not specified	40% further reduction after CRS	16.5% verified cessation at week 12	CRS behavioral adjunct enhanced outcomes
Shang et al., 2025 [57]	967 (meta-analysis)	Various	Various	MD = −2.57 (95% CI: −3.84, −1.29)	MD = −6.72 cigarettes/day	OR = 2.77 (95% CI: 1.56, 4.92)	≥20 sessions more effective; 10 Hz superior

## Data Availability

No new data were created or analyzed in this study. Data sharing is not applicable to this article.

## Supplementary Materials

The following supporting information can be downloaded at: https://www.mdpi.com/article/10.3390/brainsci16040392/s1, Table S1. Full database-specific Boolean search strings used across five databases (January 2015–January 2026)

## Abbreviations

The following abbreviations are used in this manuscript:

ACC	Anterior Cingulate Cortex
aiTBS	Accelerated Intermittent Theta Burst Stimulation
CI	Confidence Interval
ADHD	Attention Deficit/Hyperactivity Disorder
CPD	Cigarettes Per Day
CRS	Cue-Restricted Smoking
cTBS	Continuous Theta Burst Stimulation
DLPFC	Dorsolateral Prefrontal Cortex
fMRI	Functional Magnetic Resonance Imaging
FTND	Fagerström Test for Nicotine Dependence
HF-rTMS	High-frequency Repetitive Transcranial Magnetic Stimulation
IC	Inhibitory Control
IFG	Inferior Frontal Gyrus
iTBS	Intermittent Theta Burst Stimulation
ITT	Intent-to-treat
L	Left
MD	Mean Difference
MFG	Middle Frontal Gyrus
mOFC	Medial Orbitofrontal Cortex
MPH	Methylphenidate
MT	Motor Threshold
MRI	Magnetic Resonance Imaging
NAc	Nucleus Accumbens
OR	Odds Ratio
PFC	Prefrontal Cortex
PPC	Posterior Parietal Cortex
R	Right
ReHo	Regional Homogeneity
rsFC	Resting-state Functional Connectivity
rTMS	Repetitive Transcranial Magnetic Stimulation
SFG	Superior Frontal Gyrus
tDCS	Transcranial Direct Current Stimulation
TQSU	Tobacco Questionnaire for Smoking Urges
TMS	Transcranial Magnetic Stimulation
VAS	Visual Analog Scale
vmPFC	Ventromedial Prefrontal Cortex

## References

[1] World Health Organization (2021). WHO Report on the Global Tobacco Epidemic 2021: Addressing New and Emerging Products.

[2] Goodchild M., Nargis N., Tursan d’Espaignet E. (2018). Global Economic Cost of Smoking-Attributable Diseases. Tob. Control.

[3] Zainel A.A., Al Mujalli H., Yfakhroo A.I., Mohamed H.A.E., Al Nuaimi A.S., Syed M.A., Syed M.A. (2024). Investigating the Socio-Demographic Characteristics and Smoking Cessation Incidence among Smokers Accessing Smoking Cessation Services in Primary Care Settings of Qatar: A Historical Cohort Study. Discov. Public Health.

[4] Beis I., Dimou A., Kotoulas S.-C., Pataka A. (2025). Nicotine Replacement Therapy as a Smoking Cessation Tool for Adolescents: An Update. Front. Psychiatry.

[5] Rahimi F., Massoudifar A., Rahimi R. (2024). Smoking Cessation Pharmacotherapy; Varenicline or Bupropion?. DARU J. Pharm. Sci..

[6] Hartmann-Boyce J., McRobbie H., Lindson N., Bullen C., Begh R., Theodoulou A., Notley C., Rigotti N.A., Turner T., Butler A.R. (2021). Electronic Cigarettes for Smoking Cessation. Cochrane Database Syst. Rev..

[7] Livingstone-Banks J., Fanshawe T.R., Thomas K.H., Theodoulou A., Hajizadeh A., Hartman L., Lindson N. (2023). Nicotine Receptor Partial Agonists for Smoking Cessation. Cochrane Database Syst. Rev..

[8] Wittenberg R.E., Wolfman S.L., De Biasi M., Dani J.A. (2020). Nicotinic Acetylcholine Receptors and Nicotine Addiction: A Brief Introduction. Neuropharmacology.

[9] Toyoda H., Koga K. (2021). Nicotine Exposure during Adolescence Leads to Changes of Synaptic Plasticity and Intrinsic Excitability of Mice Insular Pyramidal Cells at Later Life. Int. J. Mol. Sci..

[10] Conti A.A., Tolomeo S., Baldacchino A., Steele J.D. (2024). Blunted Midbrain Reward Activation during Smoking Withdrawal: A Preliminary Study. Front. Pharmacol..

[11] Volkow N.D., Michaelides M., Baler R. (2019). The Neuroscience of Drug Reward and Addiction. Physiol. Rev..

[12] Menon V., D’Esposito M. (2022). The Role of PFC Networks in Cognitive Control and Executive Function. Neuropsychopharmacology.

[13] Friedman N.P., Robbins T.W. (2022). The Role of Prefrontal Cortex in Cognitive Control and Executive Function. Neuropsychopharmacology.

[14] Li X., Kass G., Wiers C.E., Shi Z. (2024). The Brain Salience Network at the Intersection of Pain and Substance Use Disorders: Insights from Functional Neuroimaging Research. Curr. Addict. Rep..

[15] Cocchi L., Zalesky A., Nott Z., Whybird G., Fitzgerald P.B., Breakspear M. (2018). Transcranial Magnetic Stimulation in Obsessive-Compulsive Disorder: A Focus on Network Mechanisms and State Dependence. Neuroimage Clin..

[16] El-Shahawy O., Salehi M., Saeidi M., Jaka S., Lopez J., Yakhchalian P., Abbasi-Kangevari M., Gunturu S. (2025). Exploring Applications of Transcranial Magnetic Stimulation in Child and Adolescent Psychiatry: A Narrative Review. J. Clin. Med..

[17] Siebner H.R., Funke K., Aberra A.S., Antal A., Bestmann S., Chen R., Classen J., Davare M., Di Lazzaro V., Fox P.T. (2022). Transcranial Magnetic Stimulation of the Brain: What Is Stimulated?—A Consensus and Critical Position Paper. Clin. Neurophysiol..

[18] Zhong M., Cywiak C., Metto A.C., Liu X., Qian C., Pelled G. (2021). Multi-Session Delivery of Synchronous rTMS and Sensory Stimulation Induces Long-Term Plasticity. Brain Stimul..

[19] Di Lazzaro V., Pilato F., Dileone M., Profice P., Oliviero A., Mazzone P., Insola A., Ranieri F., Tonali P.A., Rothwell J.C. (2008). Low-Frequency Repetitive Transcranial Magnetic Stimulation Suppresses Specific Excitatory Circuits in the Human Motor Cortex. J. Physiol..

[20] Luber B., Lisanby S.H. (2014). Enhancement of Human Cognitive Performance Using Transcranial Magnetic Stimulation (TMS). Neuroimage.

[21] Huang Y., Zelmann R., Hadar P., Dezha-Peralta J., Richardson R.M., Williams Z.M., Cash S.S., Keller C.J., Paulk A.C. (2024). Theta-Burst Direct Electrical Stimulation Remodels Human Brain Networks. Nat. Commun..

[22] Harmelech T., Roth Y., Tendler A. (2021). Deep TMS H7 Coil: Features, Applications and Future. Expert Rev. Med. Devices.

[23] Eichhammer P., Johann M., Kharraz A., Binder H., Pittrow D., Wodarz N., Hajak G. (2003). High-Frequency Repetitive Transcranial Magnetic Stimulation Decreases Cigarette Smoking. J. Clin. Psychiatry.

[24] Amiaz R., Levy D., Vainiger D., Grunhaus L., Zangen A. (2009). Repeated High-Frequency Transcranial Magnetic Stimulation over the Dorsolateral Prefrontal Cortex Reduces Cigarette Craving and Consumption. Addiction.

[25] Zangen A., Moshe H., Martinez D., Barnea-Ygael N., Vapnik T., Bystritsky A., Duffy W., Toder D., Casuto L., Grosz M.L. (2021). Repetitive Transcranial Magnetic Stimulation for Smoking Cessation: A Pivotal Multicenter Double-Blind Randomized Controlled Trial. World Psychiatry.

[26] Harmelech T., Hanlon C.A., Tendler A. (2023). Transcranial Magnetic Stimulation as a Tool to Promote Smoking Cessation and Decrease Drug and Alcohol Use. Brain Sci..

[27] Ibrahim C., Tang V.M., Blumberger D.M., Le Foll B. (2024). Repetitive Transcranial Magnetic Stimulation for Smoking Cessation. CMAJ.

[28] Wang T., Li R., Chen D., Xie M., Li Z., Mao H., Ling Y., Liang X., Xu G., Zhang J. (2024). Modulation of High-Frequency rTMS on Reward Circuitry in Individuals with Nicotine Dependence: A Preliminary fMRI Study. Neural Plast..

[29] Gersner R., Barnea-Ygael N., Tendler A. (2023). Moderators of the Response to Deep TMS for Smoking Addiction. Front. Psychiatry.

[30] Wills L., Ables J.L., Braunscheidel K.M., Caligiuri S.P.B., Elayouby K.S., Fillinger C., Ishikawa M., Moen J.K., Kenny P.J. (2022). Neurobiological Mechanisms of Nicotine Reward and Aversion. Pharmacol. Rev..

[31] Tiwari R.K., Sharma V., Pandey R.K., Shukla S.S. (2020). Nicotine Addiction: Neurobiology and Mechanism. J. Pharmacopunct..

[32] Addicott M.A., Sweitzer M.M., McClernon F.J. (2019). The Effects of Nicotine and Tobacco Use on Brain Reward Function: Interaction with Nicotine Dependence Severity. Nicotine Tob. Res..

[33] Lucatch A.M., Lowe D.J.E., Clark R.C., Kozak K., George T.P. (2018). Neurobiological Determinants of Tobacco Smoking in Schizophrenia. Front. Psychiatry.

[34] Flaudias V., Picot M.C., Lopez-Castroman J., Llorca P.-M., Schmitt A., Perriot J., Georgescu V., Courtet P., Quantin X., Guillaume S. (2016). Executive Functions in Tobacco Dependence: Importance of Inhibitory Capacities. PLoS ONE.

[35] Chawla M., Garrison K.A. (2018). Neurobiological Considerations for Tobacco Use Disorder. Curr. Behav. Neurosci. Rep..

[36] Soutschek A., Bulley A., Wittekind C.E. (2022). Metacognitive Deficits Are Associated with Lower Sensitivity to Preference Reversals in Nicotine Dependence. Sci. Rep..

[37] Scarlata M., Keeley R.J., Stein E.A. (2021). Nicotine Addiction: Translational Insights from Circuit Neuroscience. Pharmacol. Biochem. Behav..

[38] Viswanath H., Velasquez K.M., Thompson-Lake D.G.Y., Savjani R., Carter A.Q., Eagleman D., Baldwin P.R., De La Garza R., Salas R. (2015). Alterations in Interhemispheric Functional and Anatomical Connectivity Are Associated with Tobacco Smoking in Humans. Front. Hum. Neurosci..

[39] Shankar K., Zahedi S., George O. (2025). The Influence of Stress and Social Defeat on Neurobiological Reinforcement Mechanisms across Reward to Withdrawal in Nicotine Addiction. Psychopharmacology.

[40] Svechnikova E.V., Morzhanaeva M.A., Lemytskaya V.E., Rzhevskaya E.V., Gladko V.V., Artemyeva N.O. (2025). Therapeutic Potential of Transcranial Magnetic Stimulation in Mental Health Issues. Pharmateca.

[41] Chang D., Zhang J., Peng W., Shen Z., Gao X., Du Y., Ge Q., Song D., Shang Y., Wang Z. (2018). Smoking Cessation with 20 Hz Repetitive Transcranial Magnetic Stimulation (rTMS) Applied to Two Brain Regions: A Pilot Study. Front. Hum. Neurosci..

[42] Mahoney J.J., Hanlon C.A., Marshalek P.J., Rezai A.R., Krinke L. (2020). Transcranial Magnetic Stimulation, Deep Brain Stimulation, and Other Forms of Neuromodulation for Substance Use Disorders: Review of Modalities and Implications for Treatment. J. Neurol. Sci..

[43] Aronson Fischell S., Ross T.J., Deng Z.D., Salmeron B.J., Stein E.A. (2020). Transcranial Direct Current Stimulation Applied to the Dorsolateral and Ventromedial Prefrontal Cortices in Smokers Modifies Cognitive Circuits Implicated in the Nicotine Withdrawal Syndrome. Biol. Psychiatry Cogn. Neurosci. Neuroimaging.

[44] Sweitzer M.M., Kollins S.H., Kozink R.V., Hallyburton M., English J., Addicott M.A., Oliver J.A., McClernon F.J. (2018). ADHD, Smoking Withdrawal, and Inhibitory Control: Results of a Neuroimaging Study with Methylphenidate Challenge. Neuropsychopharmacology.

[45] Li X., Sahlem G.L., Badran B.W., McTeague L.M., Hanlon C.A., Hartwell K.J., Henderson S., George M.S. (2017). Transcranial Magnetic Stimulation of the Dorsolateral Prefrontal Cortex Inhibits Medial Orbitofrontal Activity in Smokers. Am. J. Addict..

[46] Li X., Hartwell K.J., Henderson S., Badran B.W., Brady K.T., George M.S. (2020). Two Weeks of Image-Guided Left Dorsolateral Prefrontal Cortex Repetitive Transcranial Magnetic Stimulation Improves Smoking Cessation: A Double-Blind, Sham-Controlled, Randomized Clinical Trial. Brain Stimul..

[47] Li Z., Sha X., Zhang Q., Li S., Xie M., Wang T., Chen D., Xin E., Zhang J. (2025). Repetitive Transcranial Magnetic Stimulation for Nicotine Addiction: A Regional Homogeneity Study Based on Resting-State fMRI. Psychiatry Res. Neuroimaging.

[48] Mikellides G., Michael P., Psalta L., Stefani A., Schuhmann T., Sack A.T. (2022). Accelerated Intermittent Theta Burst Stimulation in Smoking Cessation: Placebo Effects Equal to Active Stimulation When Using Advanced Placebo Coil Technology. Front. Psychiatry.

[49] Upton S., Brown A.A., Ithman M., Newman-Norlund R., Sahlem G., Prisciandaro J.J., McClure E.A., Froeliger B. (2023). Effects of Hyperdirect Pathway Theta Burst Transcranial Magnetic Stimulation on Inhibitory Control, Craving, and Smoking in Adults with Nicotine Dependence: A Double-Blind, Randomized Crossover Trial. Biol. Psychiatry Cogn. Neurosci. Neuroimaging.

[50] Shevorykin A., Carl E., Mahoney M.C., Hanlon C.A., Liskiewicz A., Rivard C., Alberico R., Belal A., Bensch L., Vantucci D. (2022). Transcranial Magnetic Stimulation for Long-Term Smoking Cessation: Preliminary Examination of Delay Discounting as a Therapeutic Target and the Effects of Intensity and Duration. Front. Hum. Neurosci..

[51] Ismail Y.A., Habib O.K., El-Banan Y., Ramadan R., Henidak M.O., El-Badry M.E., Rabea A.R., Fouad A.A., El-Tahlawy S.I., El-Shawa R.M. (2025). Efficacy of Repetitive Transcranial Magnetic Stimulation for Smoking Cessation: A Systematic Review and Meta-Analysis. Middle East Curr. Psychiatry.

[52] Mousa S.W., Badawy A.A., Attia G.F., Ghareeb W.A. (2025). Efficacy and Safety of Repetitive Transcranial Magnetic Stimulation in the Treatment of Male Smokers with Tobacco Use Disorder: A Randomized Controlled Trial. Middle East Curr. Psychiatry.

[53] Petersen N., Apostol M.R., Jordan T., Ngo T.D.P., Kearley N.W., London E.D., Leuchter A.F. (2025). Comparing Neuromodulation Targets to Reduce Cigarette Craving and Withdrawal: A Randomized Clinical Trial. Neuropsychopharmacology.

[54] Chen D., Li Z., Xie M., Wang T., Li R., Chen Y., Li S., Zhang Q., Ling Y., Liang X. (2025). Repetitive Transcranial Magnetic Stimulation Reduces Nicotine Dependence and Potentially Modulates White Matter Microstructure in Smokers: A Pilot Study by Diffusion Spectrum Imaging. Front. Neurol..

[55] Du X., Regenold W., Hong E. (2021). Improving Smoking Cessation in Schizophrenia by Functional Connectivity-Guided TMS. Brain Stimul..

[56] Scholz J.R., Bellini B.B., Ziotti S.D.V., Abe T.O., Arnaut D., Alberto R.L., Marcolin M.A., Tonstad S. (2025). Cue-Restricted Smoking as a Behavioral Adjunct for Smoking Cessation: Observational Sub-Analysis of a Randomized Trial of Deep Transcranial Magnetic Stimulation. Tob. Prev. Cessat..

[57] Shang X., Guo L., Wang Y., Guo K., Yang K. (2025). Effectiveness and Safety of Repeated Transcranial Magnetic Stimulation for Smoking Cessation: A Systematic Review and Meta-Analysis. Gen. Hosp. Psychiatry.

[58] Han X., Zhu Z., Luan J., Lv P., Xin X., Zhang X., Shmuel A., Yao Z., Ma G., Zhang B. (2023). Effects of Repetitive Transcranial Magnetic Stimulation and Their Underlying Neural Mechanisms Evaluated with Magnetic Resonance Imaging-Based Brain Connectivity Network Analyses. Eur. J. Radiol. Open.

[59] Sheffer C.E., Bickel W.K., Brandon T.H., Franck C.T., Deen D., Panissidi L., Abdali S.A., Pittman J.C., Lunden S.E., Prashad N. (2018). Preventing Relapse to Smoking with Transcranial Magnetic Stimulation: Feasibility and Potential Efficacy. Drug Alcohol Depend..

[60] Zaremehrjardi G., Ghanbari Jolfaei A., Salehian R., Ahmadkhaniha H.R. (2020). Effect of Repetitive Transcranial Magnetic Stimulation (rTMS) on Nicotine Use Disorder in Patients with Schizophrenia: A Randomized Double-Blind Clinical Trial. J. Iran. Med. Counc..

[61] Mattioli F., Maglianella V., D’Antonio S., Trimarco E., Caligiore D. (2024). Non-Invasive Brain Stimulation for Patients and Healthy Subjects: Current Challenges and Future Perspectives. J. Neurol. Sci..

[62] Koob G.F., Volkow N.D. (2016). Neurobiology of Addiction: A Neurocircuitry Analysis. Lancet Psychiatry.

[63] Liu Q., Cui H., Li J., Shen Y., Zhang L., Zheng H. (2024). Modulation of dlPFC Function and Decision-Making Capacity by Repetitive Transcranial Magnetic Stimulation in Methamphetamine Use Disorder. Transl. Psychiatry.

[64] Rabat Y., Chanraud S., Abdallah M., Sibon I., Berthoz S. (2022). Precision Preventive Medicine of Relapse in Smoking Cessation: Can MRI Inform the Search of Intermediate Phenotypes?. Biology.

[65] Fonteneau C., Merida I., Redoute J., Haesebaert F., Lancelot S., Costes N., Mondino M., Brunelin J. (2025). Modulation of Dopaminergic Transmission and Brain Activity by Frontotemporal tDCS: A Multimodal PET-MR Imaging Study. Brain Stimul..

