# Supplementary material for: Transcranial Magnetic Stimulation in Smoking Cessation: A Narrative Review of Neurobiological Mechanisms from Craving Modulation to Neural Circuit Restoration
Source: Brain Sci. 2026 Apr 2;16(4):392. doi: 10.3390/brainsci16040392 (PMC13114976; doi:10.3390/brainsci16040392)
Supplement: Supplementary file 1 [file brainsci-16-00392-s001.zip › brainsci-4192716-supplementary.pdf]

Supplementary Materials

Transcranial Magnetic Stimulation in Smoking Cessation: A Narrative Review of Neurobiological Mechanisms from Craving Modulation to Neural Circuit Restoration

Dan-Alexandru Constantin, Denisa Bianca Cristina, Florin Gabriel Leașu, Andrada-Georgiana Nacu * and Liliana Marcela Rogozea

* Correspondence: andradanacu@yahoo.com

Table S1. Full database-specific Boolean search strings used across five databases (January 2015–January 2026).

The search strategy was built around three conceptual blocks combined with the Boolean operator AND:

Block 1—Intervention: TMS modality terms

Block 2—Condition: Smoking and nicotine dependence terms

Block 3—Mechanism/Outcome: Neural, craving, and cessation outcome terms

The master string below was adapted to the syntax of each database as described in the sections that follow. All searches were limited to: (i) publications from January 2015 to January 2026; (ii) human participants; (iii) English language. Field tags, subject headings, and truncation symbols were applied as permitted by each database interface.

Master string (plain language, database-independent):

```
("transcranial magnetic stimulation" OR "repetitive transcranial magnetic
stimulation" OR "rTMS" OR "deep transcranial magnetic stimulation" OR "deep TMS"
OR "theta burst stimulation" OR "TBS" OR "intermittent theta burst stimulation"
OR "continuous theta burst stimulation" OR "iTBS" OR "cTBS")
AND
("smoking" OR "nicotine dependence" OR "nicotine addiction" OR "tobacco use
disorder" OR "tobacco use" OR "tobacco smoking" OR "cigarette smoking" OR
"smoking cessation" OR "quit smoking" OR "tobacco cessation")
AND
("craving" OR "cue reactivity" OR "functional connectivity" OR "neuroimaging" OR
"reward circuit" OR "reward circuitry" OR "prefrontal cortex" OR "dorsolateral
prefrontal cortex" OR "DLPFC" OR "neural plasticity" OR "neuroplasticity" OR
"brain stimulation" OR "neuromodulation" OR "abstinence" OR "relapse" OR "fMRI"
OR "PET" OR "brain connectivity")
```

Database	Interface / Platform	Full Search String	Filters / Limiters Applied
**PubMed/ MEDLINE**	pubmed.ncbi.nlm.nih.gov<br>*Advanced Search Builder + MeSH terms*	(("Transcranial Magnetic Stimulation"[MeSH] OR "transcranial magnetic stimulation"[tiab] OR "repetitive transcranial magnetic stimulation"[tiab]<br>OR "rTMS"[tiab] OR "deep transcranial magnetic stimulation"[tiab]<br>OR "deep TMS"[tiab] OR "theta burst stimulation"[tiab] OR "TBS"[tiab]	Date: 2015/01/01–2026/01/31<br>Species: Humans<br>Language: English<br>Article types: Clinical Trial, RCT, Meta-Analysis, Systematic Review, Observational Study, Neuroimaging Study (not restricted — all types screened)

		OR "iTBS"[tiab] OR "cTBS"[tiab])<br>AND<br>("Tobacco Use Disorder"[MeSH] OR "Smoking Cessation"[MeSH]<br>OR "Tobacco Smoking"[MeSH] OR "Nicotine Dependence"[tiab]<br>OR "smoking cessation"[tiab] OR "nicotine addiction"[tiab]<br>OR "tobacco use"[tiab] OR "cigarette smoking"[tiab])<br>AND<br>("craving"[tiab] OR "cue reactivity"[tiab] OR "functional<br>connectivity"[tiab] OR "neuroimaging"[tiab] OR "reward circuit*"[tiab]<br>OR "prefrontal cortex"[tiab] OR "dorsolateral prefrontal cortex"[tiab]<br>OR "DLPFC"[tiab] OR "neural plasticity"[tiab] OR "neuroplasticity"[tiab]<br>OR "abstinence"[tiab] OR "relapse"[tiab] OR "fMRI"[tiab]<br>OR "brain connectivity"[tiab] OR "neuromodulation"[tiab]))	
**Embase**	Elsevier Embase.com<br>*Emtree headings + free text (ti,ab)*	('transcranial magnetic stimulation'/exp OR 'transcranial magnetic<br>stimulation':ti,ab OR 'repetitive transcranial magnetic<br>stimulation':ti,ab OR 'rTMS':ti,ab OR 'deep transcranial magnetic<br>stimulation':ti,ab OR 'theta burst stimulation':ti,ab<br>OR 'iTBS':ti,ab OR 'cTBS':ti,ab)<br>AND<br>('smoking cessation'/exp OR 'nicotine dependence'/exp<br>OR 'tobacco use disorder'/exp OR 'nicotine addiction':ti,ab<br>OR 'cigarette smoking':ti,ab OR 'tobacco use':ti,ab<br>OR 'smoking cessation':ti,ab)<br>AND<br>('craving':ti,ab OR 'cue reactivity':ti,ab<br>OR 'functional connectivity':ti,ab OR 'neuroimaging':ti,ab<br>OR 'reward circuit*':ti,ab OR 'prefrontal cortex':ti,ab<br>OR 'dorsolateral prefrontal cortex':ti,ab OR 'DLPFC':ti,ab	Date: 2015–2026 [py]<br>Population: [humans]/lim<br>Language: [english]/lim<br>Emtree MeSH equivalents used where available for all three concept blocks

		OR 'neural plasticity':ti,ab OR 'neuroplasticity':ti,ab<br>OR 'abstinence':ti,ab OR 'relapse':ti,ab OR 'fMRI':ti,ab<br>OR 'neuromodulation':ti,ab OR 'brain connectivity':ti,ab)<br>AND [humans]/lim AND [english]/lim AND [2015-2026]/py	
**Web of Science Core Collection**	Clarivate Web of Science<br>*Topic (TS=) field search; no controlled vocabulary*	TS=("transcranial magnetic stimulation" OR "repetitive transcranial<br>magnetic stimulation" OR "rTMS" OR "deep transcranial magnetic<br>stimulation" OR "theta burst stimulation" OR "iTBS" OR "cTBS")<br>AND<br>TS=("smoking" OR "nicotine dependence" OR "nicotine addiction"<br>OR "tobacco use disorder" OR "tobacco smoking" OR "cigarette smoking"<br>OR "smoking cessation" OR "tobacco cessation")<br>AND<br>TS=("craving" OR "cue reactivity" OR "functional connectivity"<br>OR "neuroimaging" OR "reward circuit*" OR "prefrontal cortex"<br>OR "dorsolateral prefrontal cortex" OR "DLPFC"<br>OR "neural plasticity" OR "neuroplasticity" OR "abstinence"<br>OR "relapse" OR "fMRI" OR "neuromodulation"<br>OR "brain connectivity")<br>Refined by: Publication Years: 2015–2026;<br>Research Areas: Neurosciences, Psychiatry, Substance Abuse,<br>Psychology; Document Types: Article, Review	Date: Publication Years 2015–2026<br>Research Areas filter: Neurosciences; Psychiatry; Substance Abuse; Psychology; Clinical Neurology<br>Document types: Article, Review (editorials and letters excluded at screening stage)
**PsycINFO**	APA PsycINFO via APA PsycNet / EBSCO<br>*APA Thesaurus terms + free text (ti, ab)*	(DE "Transcranial Magnetic Stimulation" OR<br>TI "transcranial magnetic stimulation" OR<br>AB "transcranial magnetic stimulation" OR<br>TI "rTMS" OR AB "rTMS" OR<br>TI "deep transcranial magnetic stimulation" OR<br>AB "deep transcranial magnetic stimulation" OR	Date: 20150101–20260131<br>Population: Human<br>Language: English<br>APA Thesaurus controlled vocabulary (DE field) used for primary concepts; ti/ab free-text for modality variants

		TI "theta burst stimulation" OR AB "theta burst stimulation" OR<br>TI "iTBS" OR AB "iTBS" OR TI "cTBS" OR AB "cTBS")<br>AND<br>(DE "Smoking Cessation" OR DE "Nicotine" OR<br>DE "Tobacco Smoking" OR<br>TI "smoking cessation" OR AB "smoking cessation" OR<br>TI "nicotine dependence" OR AB "nicotine dependence" OR<br>TI "tobacco use disorder" OR AB "tobacco use disorder" OR<br>TI "cigarette smoking" OR AB "cigarette smoking")<br>AND<br>(TI "craving" OR AB "craving" OR<br>TI "functional connectivity" OR AB "functional connectivity" OR<br>TI "neuroimaging" OR AB "neuroimaging" OR<br>TI "reward circuit*" OR AB "reward circuit*" OR<br>TI "prefrontal cortex" OR AB "prefrontal cortex" OR<br>TI "DLPFC" OR AB "DLPFC" OR<br>TI "neural plasticity" OR AB "neural plasticity" OR<br>TI "abstinence" OR AB "abstinence" OR<br>TI "neuromodulation" OR AB "neuromodulation" OR<br>TI "fMRI" OR AB "fMRI")<br>Limiters: Publication Date: 20150101-20260131;<br>Language: English; Population Group: Human	
**Cochrane Central Register of Controlled Trials (CENTRAL)**	Cochrane Library (Wiley)<br>*Title, Abstract, Keywords (Word variations on)*	#1 MeSH descriptor: [Transcranial Magnetic Stimulation]<br>explode all trees<br>#2 "transcranial magnetic stimulation":ti,ab,kw<br>#3 "repetitive transcranial magnetic stimulation":ti,ab,kw<br>#4 rTMS:ti,ab,kw<br>#5 "deep transcranial magnetic stimulation":ti,ab,kw<br>#6 "theta burst stimulation":ti,ab,kw<br>#7 iTBS:ti,ab,kw<br>#8 cTBS:ti,ab,kw	Date: Jan 2015 – Jan 2026<br>CENTRAL searched for controlled trials only (by database scope)<br>MeSH "explode all trees" applied for primary clinical concepts<br>Word variations: ON (for all ti,ab,kw terms)

		#9 #1 OR #2 OR #3 OR #4 OR #5 OR #6 OR #7 OR #8<br><br>#10 MeSH descriptor: [Tobacco Use Disorder] explode all trees<br>#11 MeSH descriptor: [Smoking Cessation] explode all trees<br>#12 MeSH descriptor: [Tobacco Smoking] explode all trees<br>#13 "nicotine dependence":ti,ab,kw<br>#14 "smoking cessation":ti,ab,kw<br>#15 "cigarette smoking":ti,ab,kw<br>#16 "tobacco use":ti,ab,kw<br>#17 #10 OR #11 OR #12 OR #13 OR #14 OR #15 OR #16<br><br>#18 "craving":ti,ab,kw<br>#19 "functional connectivity":ti,ab,kw<br>#20 "neuroimaging":ti,ab,kw<br>#21 "reward circuit*":ti,ab,kw<br>#22 "prefrontal cortex":ti,ab,kw<br>#23 "DLPFC":ti,ab,kw<br>#24 "neural plasticity":ti,ab,kw<br>#25 "neuroplasticity":ti,ab,kw<br>#26 "abstinence":ti,ab,kw<br>#27 "neuromodulation":ti,ab,kw<br>#28 "fMRI":ti,ab,kw<br>#29 "brain connectivity":ti,ab,kw<br>#30 #18 OR #19 OR #20 OR #21 OR #22 OR #23 OR #24 OR #25 OR #26 OR #27 OR #28 OR #29<br><br>#31 #9 AND #17 AND #30<br>Filter: Publication date from Jan 2015 to Jan 2026	

Notes on Search Strategy

Date range: January 1, 2015 to January 31, 2026 for all databases. The lower bound was selected to focus the review on the contemporary evidence base that followed the publication of pivotal early trials and the emergence of deep TMS and theta burst stimulation protocols. Older foundational studies (e.g., Eichhammer et al. 2003; Amiaz et al. 2009) were identified through reference snowballing and cited where directly relevant to the historical rationale.

Reference snowballing: Reference lists of all included studies and of identified meta-analyses (Ismail et al. 2025; Shang et al. 2025) were hand-searched to identify additional eligible studies not captured by database searches. Snowballing was performed by two authors independently.

Deduplication: Records from all five databases were exported to a reference manager (Mendeley/Zotero). Duplicates were identified by DOI and title matching and removed prior to screening. A total of 847 unique records remained after deduplication.

Screening: Title and abstract screening was conducted independently by two authors (D-A.C. and A-G.N.) against the predefined eligibility criteria. Full texts were obtained for all records passing title/abstract screening. Full-text assessment was also performed independently by two authors, with disagreements resolved by consensus or adjudication by a third author (L-M.R.).

Total included studies: 34 studies met all eligibility criteria and were included in the narrative synthesis.

Language restriction: English only. Non-English language records were excluded at screening.

Exclusion of grey literature: Conference abstracts, unpublished trial registrations, and preprints not subsequently published in peer-reviewed journals were excluded.
